# Multi-Omic Profiling of Multi-Biosamples Reveals the Role of Amino Acid and Nucleotide Metabolism in Endometrial Cancer

**DOI:** 10.3389/fonc.2022.861142

**Published:** 2022-04-29

**Authors:** Runqiu Yi, Liying Xie, Xiaoqing Wang, Chengpin Shen, Xiaojun Chen, Liang Qiao

**Affiliations:** ^1^ Department of Chemistry, Shanghai Stomatological Hospital, and Obstetrics and Gynecology Hospital of Fudan University, Fudan University, Shanghai, China; ^2^ Shanghai Omicsolution Co., Ltd., Shanghai, China

**Keywords:** endometrial cancer, biomarkers, metabolic pathways, metabolomics, proteomics

## Abstract

**Background:**

Endometrial cancer (EC) is one of the most common gynecological cancers. The traditional diagnosis of EC relies on histopathology, which, however, is invasive and may arouse tumor spread. There have been many studies aiming to find the metabolomic biomarkers of EC to improve the early diagnosis of cancer in a non-invasive or minimally invasive way, which can also provide valuable information for understanding the disease. However, most of these studies only analyze a single type of sample by metabolomics, and cannot provide a comprehensive view of the altered metabolism in EC patients. Our study tries to gain a pathway-based view of multiple types of samples for understanding metabolomic disorders in EC by combining metabolomics and proteomics.

**Methods:**

Forty-four EC patients and forty-three controls were recruited for the research. We collected endometrial tissue, urine, and intrauterine brushing samples. Untargeted metabolomics and untargeted proteomics were both performed on the endometrial tissue samples, while only untargeted metabolomics was performed on the urine and intrauterine brushing samples.

**Results:**

By integrating the differential metabolites and proteins between EC patients and controls detected in the endometrial tissue samples, we identified several EC-related significant pathways, such as amino acid metabolism and nucleotide metabolism. The significance of these pathways and the potential of metabolite biomarker-based diagnosis were then further verified by using urine and intrauterine brushing samples. It was found that the regulation of metabolites involved in the significant pathways showed similar trends in the intrauterine brushings and the endometrial tissue samples, while opposite trends in the urine and the endometrial tissue samples.

**Conclusions:**

With multi-omics characterization of multi-biosamples, the metabolomic changes related to EC are illustrated in a pathway-based way. The network of altered metabolites and related proteins provides a comprehensive view of altered metabolism in the endometrial tissue samples. The verification of these critical pathways by using urine and intrauterine brushing samples provides evidence for the possible non-invasive or minimally invasive biopsy for EC diagnosis in the future.

## Introduction

Endometrial cancer (EC) is one of the most common cancers among women in the world. According to the latest statistics, EC accounted for 417,367 new cases and 97,370 deaths in 2020 worldwide (1). Risk factors like obesity (2), diabetes (3), and hypertension (4) have been found to relate to the occurrence and deterioration of EC, but the pathogenesis of EC is still unclear. Histopathology is the gold standard for tumor diagnosis, but is less efficient in the detection of small lesions (5). Moreover, the traditional histopathology methods require complex operations, which are highly invasive and may arouse tumor spread (6). Finding biomarkers for EC can support early screening, diagnosis, or postoperative follow-up in a non-invasive or minimally invasive way. It has been reported that the increase of serum cancer antigen 125 (CA125) is a sign of several types of cancers including EC (7), but is not specific for any of the cancers. The lack of specific screening methods, the lack of non-invasive diagnostic methods, and the lack of comprehensive understanding of pathogenesis for EC are the major current problems in the study, detection, and treatment of EC.

The quantitative characterization of metabolites involved in various metabolism pathways can reveal the dynamic status of investigated systems, and provide opportunities for finding disease biomarkers and investigating disease mechanisms (8). Previous studies on EC metabolomics mainly measured a single type of biosample, such as tissue (9, 10), plasma (11–16), serum (15, 17–24), urine (25), and cervicovaginal fluids (26), focusing on the up- or down-regulation of specific compounds or the selection of a group of compounds for building diagnostic models. Though many promising results have been achieved in identifying metabolite biomarkers of EC, especially lipids, hormones, and amino acids (27), the inconsistency among various biosamples in the studies was not taken into consideration, and it is still unclear how metabolomic pathways are perturbed in EC (28, 29). Since metabolites are the very downstream compounds in the metabolic process and one metabolite may participate in several reactions, the dysregulation of a specific metabolite may result from various processes, making it difficult to identify the real alteration of metabolic pathways in EC solely by metabolomic analysis. The current limitation of untargeted metabolomics on compound identification (30) requires the utilization of other techniques, e.g., proteomics, to fetch up. As demonstrated in studies on other diseases like COVID-19, multi-omics analyses can facilitate the understanding of metabolic changes related to pathogenesis (31), and multi-organ analyses can provide a comprehensive landscape of the corresponding disease (32).

In this work, we performed multi-omics analysis for characterizing multiple types of clinical samples to study the perturbation of metabolomic pathways in EC. By integrating metabolomics with proteomics, a more credible explanation for the metabolic dysregulation of EC was achieved in a pathway-based way. By combining and comparing the results of multi-biosamples, the selected dysregulated pathways were further verified, and the potential of non-invasive or minimal invasive diagnosis of EC based on metabolite biomarkers was assessed. Forty-four EC patients and forty-three controls were recruited for this research. The endometrial tissue, urine, and intrauterine brushing samples were collected for proteomic and metabolomic analysis. Intrauterine brushings are bioliquid samples collected by aspiration biopsy using special brushes, containing a mixture of endometrial cells, blood cells, and surrounding secretion. Based on the differential metabolites and proteins between EC patients and controls detected in the endometrial tissue samples, EC-related significant pathways, such as amino acid metabolism and nucleotide metabolism, were identified. Then, the significance of the pathways was further evaluated using the urine and intrauterine brushing samples. The up- and down-regulation of the differential metabolites were compared among tissue, urine, and intrauterine brushing samples to illustrate the diversity of metabolism in multi-biosamples. The regulation of metabolites in the intrauterine brushings showed similar trends to that in the endometrial tissue, while the regulation of metabolites in the urine showed opposite trends compared to the tissue. We also demonstrated the potential of non-invasive or minimally invasive biopsy for EC diagnosis using the identified metabolic biomarkers with urine or intrauterine brushing samples.

## Materials and Methods

### Chemicals

Acetonitrile (ACN), formic acid (FA), methanol, and deionized water were all HPLC grade, from Merck (Darmstadt, Germany). Phosphate buffered saline (PBS) and sodium dodecyl sulfate (SDS) were from Solarbio (Beijing, China). Analytical reagent grade acetone was from Sinopharm (Shanghai, China). Iodoacetamide (IAA), trizma base, urea, and C18 ZipTip were from Sigma-Aldrich (Darmstadt, Germany). Proteome grade trypsin was from Promega (Madison, WI, USA). Bond-breaker TCEP solution (0.5 M), triethylammonium bicarbonate (TEAB), protease inhibitor cocktail (EDTA-free, 100X), Pierce enhanced bicinchoninic acid (BCA) protein assay kit, and Pierce quantitative colorimetric peptide assay kit (23275) were from Thermo Fisher Scientific (San Jose, CA, USA).

### Clinical Sample Collection and Preparation

The enrolled EC patients were all suffering from type I endometrial carcinoma, or more specifically, grade 1 and grade 2 endometrioid endometrial carcinoma. The enrolled controls all had a normal state of endometrium, but suffered from gynecological diseases including hysteromyoma, cyst, endometrial polyps, and cervix diseases.

Endometrial tissues were collected after surgical intervention. Each tissue sample (about 50 mg) was placed in a sterile container, properly labeled, and stored at −80°C immediately after sample collection. Urine specimens were collected in the morning before the day of the surgical operation and after the subjects had fasted for 10–12 h (33). The second micturition was collected for each subject and aliquots (about 5 ml) were stored at −80°C. Intrauterine brushings were collected using a special hollow tube with a brush, as a mixture of endometrial cells, blood cells, and surrounding secretion by aspiration biopsy. The mixture was then added with 1 ml of ice-cold 80% methanol/water immediately and stored at −80°C.

### Metabolite Extraction

Ice-cold 80% methanol/water was used as extracting solution to extract metabolites. Concretely, 50 mg of thawed tissue sample was added with 1 ml extracting solution and then ground thoroughly. Thawed urine (200 μl) was added with 800 μl of extracting solution and vortexed for 15 s. The processed tissue and urine samples were stored at −80°C overnight for a thorough extraction of metabolites. Intrauterine brushings had already been added with the extracting solution during collection and stored at −80°C before further steps. All the liquid mixtures of different biosamples were then thawed and centrifuged (12,000 rpm, 5 min, 4°C). The supernatant was lyophilized and stored at −80°C until measurement.

### Protein Extraction, Digestion, and Quantification

Tissue samples were rinsed by PBS, ground thoroughly, and resuspended in a lysis solution (8 μl per 1-mg sample) containing 1% SDS, 8 M urea, and 1× protease inhibitor cocktail in deionized water. Samples were then sonicated for 30 min in an ice-water bath using an ultrasonic cell homogenizer (Ningbo Scientz Biotechnology, Ningbo, China) with the working power ≤ 47.5 W to avoid bubble formation. Protein extracts were obtained after centrifugation (15,000 rpm, 15 min, 4°C) and the protein level in the supernatant was determined by the Pierce BCA protein assay kit. One hundred micrograms of protein per sample was transferred into a new centrifuge tube, and the final volume was adjusted to 100 μl with 8 M urea. Two microliters of 0.5 M TCEP was added and the sample was incubated at 37°C for 1 h, and then 4 μl of 1 M IAA was added to the sample and the incubation lasted for 40 min protected from light at room temperature. After that, five volumes of −20°C pre-chilled acetone was added to precipitate the proteins overnight at −20°C. The precipitates were washed twice with 1 ml of pre-chilled 90% acetone aqueous solution and then re-dissolved in 100 μl of 100 mM TEAB. Proteome grade modified trypsin was added at the ratio of 1:50 (enzyme:protein, weight:weight) to digest the proteins at 37°C overnight. The peptide mixture was desalted by C18 ZipTip, quantified by Pierce quantitative colorimetric peptide assay, and then lyophilized.

### LC-MS/MS Analysis

For untargeted metabolomic analysis, three replicated injections were performed for each sample. The metabolites were analyzed by an ESI-Q-TOF mass spectrometer (SCIEX TripleTOF 4600, USA) coupled with an LC-20A HPLC system (Shimadzu, Tokyo, Japan). Each lyophilized sample was re-dissolved in 100 μl of 95% solvent A (0.1% FA in water) and centrifuged (8,000 rpm, 20 min, 4°C) to remove the insoluble constituents. Five microliters of the extracted metabolite sample were loaded by an autosampler, and the metabolites were separated by a Waters ACQUITY UPLC HSS T3 C18 column (100 × 2.1 mm, 1.8 μm, Waters, Milford, MA, USA) with the flow rate of 0.2 ml/min. Water (containing 0.1% FA) and ACN were used as solvent A and B, respectively, with the gradient elution program as follows: 0–6–11–13–15–20–30–30–40 min, 5%–25%–35%–40%–55%–95%–95%–5%–5% of solvent B. The ESI-Q-TOF was run in information-dependent acquisition (IDA) mode with parameters optimized as follows: (1) MS: ion spray voltage = +5,500 V; scan range = 50–1,000 m/z; precursor ions = 15; excluding precursor for 3 s; enabling dynamic background subtraction; (2) MS/MS: collision energy = 45 eV.

For untargeted proteomic analysis, the peptides were re-dissolved in solvent A (0.1% FA in water) to reach the concentration of 0.5 μg/μl and analyzed by online nanospray LC-MS/MS with an Orbitrap Fusion™ Lumos™ Tribrid™ mass spectrometer (Thermo Fisher Scientific, MA, USA) coupled to an EASY-nanoLC 1200 system (Thermo Fisher Scientific, MA, USA). The peptide sample (3 μl) was loaded onto an analytical column (Acclaim PepMap C18, 75 μm x 25 cm) and separated with a 120-min gradient. The column flow rate was maintained at 600 nl/min with a column temperature of 40°C. Water and ACN (both containing 0.1% FA) were used as solvent A and B, respectively, with the gradient elution program as follows: 0–4–80–110–112–120 min, 4%–7%–20%–30%–90%–90% of solvent B. The electrospray voltage of 2 kV versus the inlet of the mass spectrometer was used. The mass spectrometer was run under data independent acquisition mode and automatically switched between the MS and MS/MS modes. The parameters were as follows: (1) MS: scan range (m/z) = 350–1500; resolution = 120,000; AGC target = 4e5; maximum injection time = 50 ms; (2) HCD-MS/MS: resolution = 30,000; AGC target = 2e5; collision energy = 32; (3) DIA: variable isolation window; each window overlapping 1 m/z; window number = 60.

Different strategies were utilized for the quality control (QC) of untargeted metabolomic and proteomic analyses. For metabolomic analysis, a mixed QC sample by taking a small volume of each experimental sample served as a technical replicate throughout the data acquisition in three respective batches (tissue, urine, and intrauterine brushings). The EC and control samples were analyzed alternately in a randomized order with 3 replicates of each sample, while QC samples were injected at the beginning of each analytical batch, every 6 samples, and at the end of each analytical batch.

For proteomic analysis, QuiC (Biognosys AG, Switzerland) was used to evaluate MS stability. Full peak width at half maximum (FWHM), retention time (RT), and peak capacity of LC, as well as MS1 area, MS1/MS2 mass accuracy, MS1/MS2 scan intensity, and TIC of MS were calculated to assess the stability of measurement. Coefficient of variation, data completeness, heatmap of intensity, and consistency of identification were visualized to demonstrate the quality of the data.

### Data Analysis

For untargeted metabolomic analysis, raw data were converted to mzXML by MSConvert software (34) and then processed with R package XCMS (35). The retention time range for extraction was set as 0–20 min. The generated matrices of mass spectral features included information on m/z value, retention time, and peak intensity. MS1 signal intensities were then normalized by the summation of all peaks for an individual sample to calculate relative quantity and were performed with log transformation and auto scaling to form the matrices for subsequent statistical analysis. Univariate and multivariate statistical analyses were done by MetaboAnalyst 5.0 (http://www.metaboanalyst.ca) (36). VIP values in PLS-DA models and the *p*-values from *t*-tests on the normalized peak intensities were used to select differential features, the rule of which was VIP > 1 or *p* < 0.05. The structural identification of differential metabolites was performed by MetDNA (http://metdna.zhulab.cn/) (37), including accurate mass, MS/MS spectra, and online databases: METLIN (http://www.metlin.scripps.edu) (38).

For untargeted proteomic analysis, raw data of DIA were processed and analyzed by Spectronaut 14 (Biognosys AG, Switzerland) with default settings, and the retention time prediction type was set to dynamic iRT. Data extraction was determined based on extensive mass calibration. The ideal extraction window was determined dynamically depending on iRT calibration and gradient stability. *Q*-value (FDR) cutoff on precursor and protein level was applied as 1%. Decoy generation was set to mutate. All selected precursors passing the filters were used for quantification. MS2 interference will remove all interfering fragment ions except for the 3 least interfering ones. The average of the top 3 filtered peptides, which passed the 1% *Q*-value (FDR) cutoffs, was used as the major group quantities. The quantitative data were local normalized before statistical analysis. After Welch’s ANOVA test, differently expressed proteins were filtered with *p*.adj value < 0.05 and fold change > 1.5.

Multivariant statistical analyses, e.g., principal component analysis (PCA) and partial least squares-discriminant analysis (PLS-DA), of metabolomic and proteomic data were performed using MetaboAnalyst 5.0. Pathway analysis and enrichment analysis of metabolomic data were performed using MetaboAnalyst 5.0. Functional annotation of proteins was carried out based on the euKaryotic orthologous groups of proteins (KOG) database and Gene Ontology (GO) annotations (https://www.ebi.ac.uk/QuickGO/). Network analysis of differential metabolites and differential proteins was performed using MetaboAnalyst 5.0 based on the search tool for interactions of chemicals (STITCH) (39) and the network diagram was generated by Cytoscape 3.9.1 (40).

Metabolomic biomarker analysis was performed using MetaboAnalyst 5.0. Multivariate exploratory analysis was utilized to test the performance of models by ROC curve analyses based on the PLS-DA algorithm. ROC curves were generated by MCCV. In each MCCV, two-thirds of the samples were used to evaluate the feature importance. The top 2, 3, 5, 10…100 (max) important features were then used to build classification models that were validated for 1/3 of the samples that were left out. The procedure was repeated multiple times to calculate the performance and confidence interval of each model.

## Results

**Figure 1:**
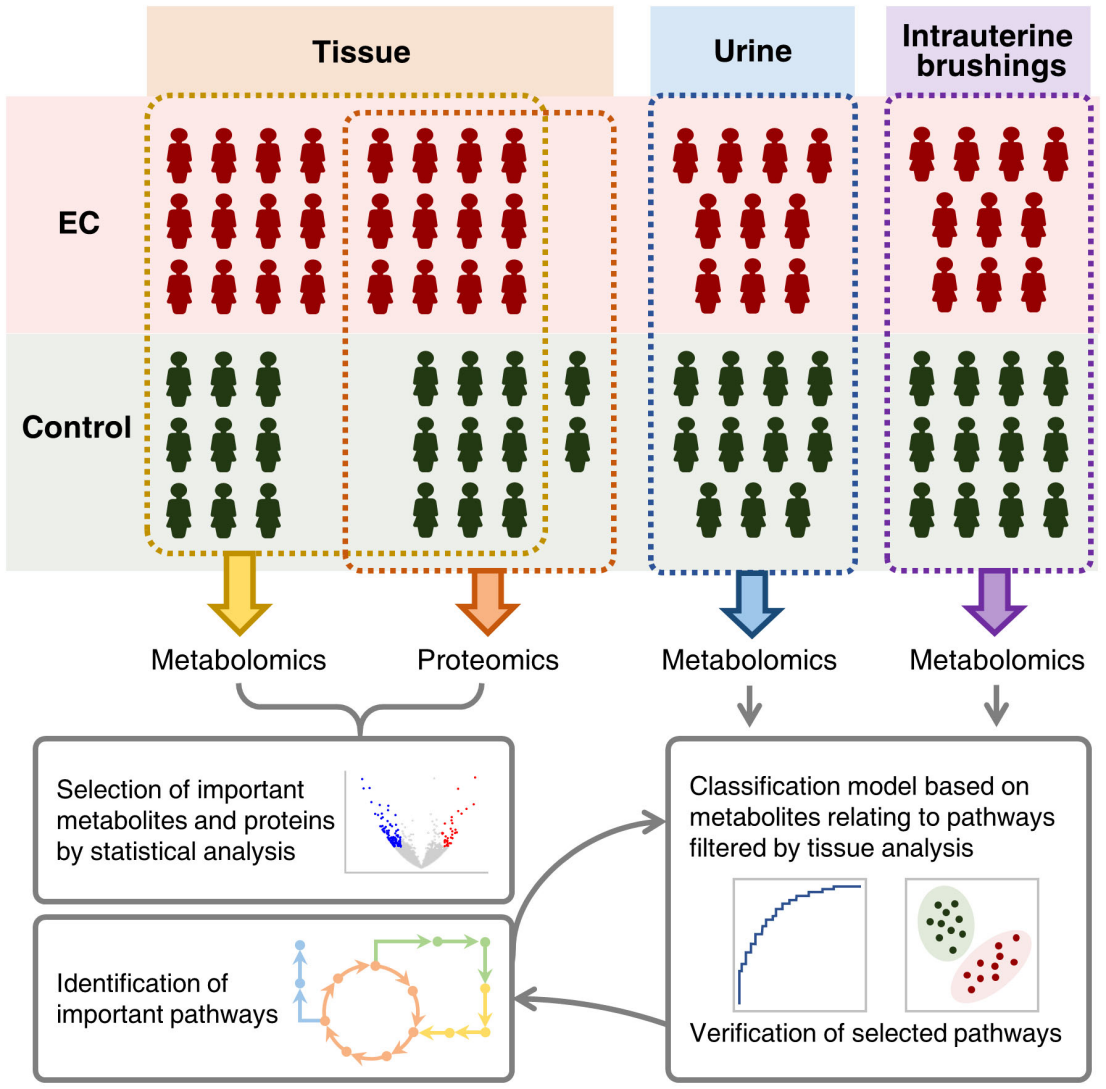
Schematic illustration of integrating metabolomic and proteomic characterization of multi-biosamples from endometrial cancer patients to identify and verify metabolomic pathways significant to the pathogenesis of endometrial cancer.

In this study, 44 patients suffering from EC and 43 controls with a normal state of endometrium were enrolled, and samples of endometrial tissue, urine, and intrauterine brushings were collected for metabolomic and proteomic analysis. The type of EC for all the patients was type I endometrial carcinoma, namely endometrioid endometrial carcinoma grade 1 (G1) and grade 2 (G2), which was estrogen dependent and closely related to metabolic processes (41). The experimental design is illustrated in **Figure 1**. Sampling details and patient information including age, body mass index (BMI), menopausal status, previous pregnancy circumstances, medical history of diabetes and hypertension, smoking history, hormone replacement therapy (HRT) history, and the grade and FIGO stage are described in the “Materials and Methods” section and **Supplementary Table 1**.

### Untargeted Metabolomic and Proteomic Profiling of EC Tissue Samples

**Figure 2:**
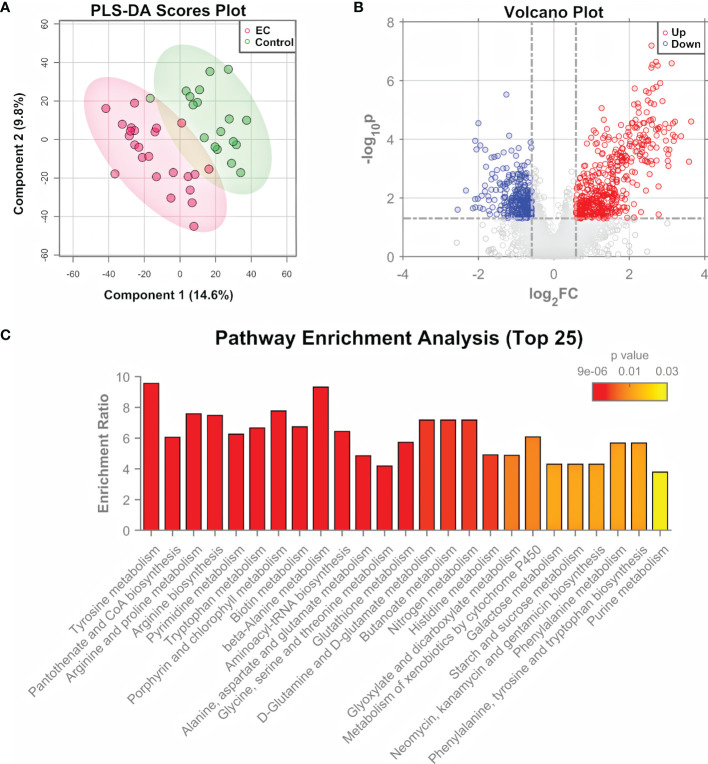
Metabolomic analysis of EC tissue samples. **(A)** PLS-DA score plot for metabolomic data of tissue samples collected from EC and control groups by LC-MS/MS in the positive ion mode. **(B)** Volcano plots of features from the metabolomic data with log_2_(FC) as the horizontal axis and −log_10_(*p*-value) as the vertical axis. FC, fold change of EC to control. **(C)** Pathway enrichment analysis of differential metabolites identified from the tissue samples of EC and control groups, showing the results of the top 25 enriched metabolomic pathways.

The significance of tumor tissues in EC pathogenesis research has been demonstrated in previous studies (9, 10), and the histopathological examination of tumor tissues is the “gold standard” of clinical diagnosis of EC (7). Tissue samples were obtained from 24 patients with EC and 18 controls, and measured by both untargeted metabolomics and proteomics for the determination of important pathways. Untargeted metabolomics was performed using an HPLC-QTOF-MS/MS system in the positive ion mode. Total ion-current chromatograms (TICs) of QC samples (**Supplementary Figure 1**) showed a good overlap, demonstrating the stability and repeatability of the measuring system. A total of 4410 features were extracted from the raw data using XCMS (35). After data normalization and transformation, PCA and PLS-DA were performed on the metabolomic data. Results showed that the EC group and control group can hardly be separated by the unsupervised analysis, i.e., PCA (**Supplementary Figure 2**), but can be clustered into two discriminative groups by the supervised analysis, i.e., PLS-DA (**Figure 2A**). Hierarchical clustering heatmap generated using the top 500 features with smallest *p*-values (**Supplementary Figure 3**) also showed that the samples from the EC group and control group bear a trend to be distinguished, but a minority of them were wrongly classified. The results indicated that the metabolomic characteristics between the EC and the control groups were generally similar, but with changes in specific features that might be derived from the metabolomic perturbation in EC. Volcano plot (**Figure 2B**) showed that many features were up- or down-regulated in the EC group compared to the control group.

The differential features were then subjected to structure identification by MetDNA (37). A total of 74 metabolites were identified from the features with *t*-test *p*-values < 0.05 or PLS-DA variable importance in the projection (VIP) values > 1 (**Supplementary Dataset 1**). In order to characterize the roles of the differential metabolites, a Kyoto Encyclopedia of Genes and Genomes (KEGG) pathway analysis was performed using the MetaboAnalyst 5.0 (36). **Figure 2C** shows the top 25 enriched pathways. The highly enriched pathways for the differential metabolites from EC tissue samples include the amino acid metabolism pathways (such as metabolism related with tyrosine, arginine, proline, and alanine), nucleotide metabolism pathways (pyrimidine metabolism and purine metabolism), and metabolism pathways of cofactors and vitamins (such as CoA biosynthesis and biotin synthesis), most of which are associated with energy metabolism (42).

**Figure 3:**
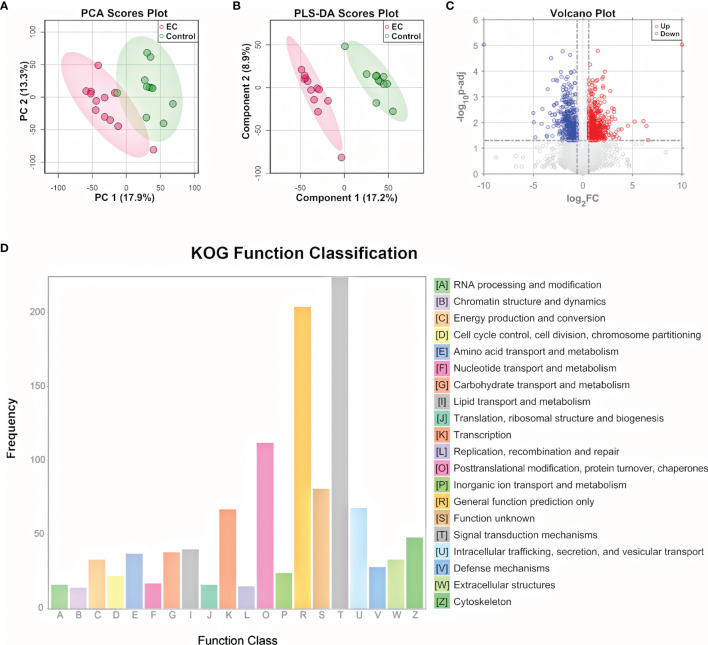
Proteomic analysis of EC tissue samples. **(A)** PCA and **(B)** PLS-DA score plot for proteomic data of tissue samples from EC and control groups by LC-MS/MS in the positive ion mode. **(C)** Volcano plots of proteins with log_2_(FC) as the horizontal axis and −log_10_(*p*-value) as the vertical axis. FC, fold change of EC to control. **(D)** KOG functional annotation of the 1,445 proteins with significant variations in EC tissue samples compared to controls.

To further explore the metabolomic changes of EC, a subset of the tissue samples with 2 new controls, i.e., 12 EC and 11 controls, were subjected to untargeted proteomic analysis, using a nano-UHPLC-Orbitrap-MS/MS system in the positive ion mode. A total of 9,042 proteins were identified and quantified by combining the EC and control groups. The heatmap containing the intensity information and clustering results of the protein groups (**Supplementary Figure 4**) showed a high consistency among samples as well as a rough division between the EC and control groups, suggesting the stability of the system and the credibility of the data. PCA and PLS-DA were then performed on the proteome data. Results showed that the EC and control groups can be well clustered into two discriminative groups by score plots of both PCA (**Figure 3A**) and PLS-DA (**Figure 3B**), indicating that the proteomic state of EC patient tissue samples was significantly perturbed compared with the control ones. Volcano plot (**Figure 3C**) showed that there were 1,445 proteins (**Supplementary Dataset 2**) significantly up- or downregulated in the EC group compared to the control group (*p*.adj values < 0.05 and FC > 1.5).

To gain a deeper understanding of the significantly changed proteins, the KOG database was applied for functional analysis. According to the annotation results of KOG (**Figure 3D**), the differential proteins were mostly distributed into 4 functional groups: signal transduction mechanisms [T]; posttranslational modification, protein turnover, and chaperones [O]; intracellular trafficking, secretion, and vesicular transport [U]; and transcription [K]. In addition to the 4 most significant functional groups, several functional groups associated with energy metabolism were also found to be prominent here, including the following: lipid transport and metabolism [I], carbohydrate transport and metabolism [G], amino acid transport and metabolism [E], nucleotide transport and metabolism [F], and energy production and conversion [C]. Pathway analysis of the differential metabolites and functional analysis of the differential proteins both emphasized the role of the bioenergetic process, especially those relating to amino acids and nucleotides, showing consistency between the metabolome and proteome data. Other bioinformatics analyses based on the differential proteins, including GO functional analysis and KEGG pathway enrichment analysis, are shown in **Supplementary Figures 5**, **6**. The GO classification indicates that genes relating to the response to hormones were highly annotated, indicating a change in the hormone state of EC (43). KEGG pathway plot showed that half of the top 20 enriched pathways were related to human diseases, which was well correlated with the fact that the samples were cancer-oriented.

### Pathway Analysis Integrating Metabolomic and Proteomic Data of Tissue Samples

To find the relation between the metabolomic and proteomic data of tissue samples, network analysis was performed for the differential metabolites and differential proteins according to their chemical structures and molecular activities. The network diagram (**Supplementary Figure 7**) showed a network of 28 metabolites and 135 proteins with 212 connections, from which the nodes highly connected to others can be seen. Among them, glutamate, dopamine, noradrenaline, adenosine 5’-monophosphate (AMP), and guanosine 5’-monophosphate (GMP) were the major centers of sub-networks. Meanwhile, the sub-networks of dopamine and noradrenaline shared overlap of some nodes, and the same occurred for the sub-networks of AMP and GMP. Glutamate, dopamine, and noradrenaline are critical intermediates in amino acid metabolism, while AMP and GMP play critical roles in ribonucleotide biosynthesis of purine metabolism. Incorporating the network analysis results with the pathway enrichment results of differential metabolites and the function classification results of differential proteins, we further focused on the pathways of amino acid metabolism and nucleotide metabolism.

**Figure 4:**
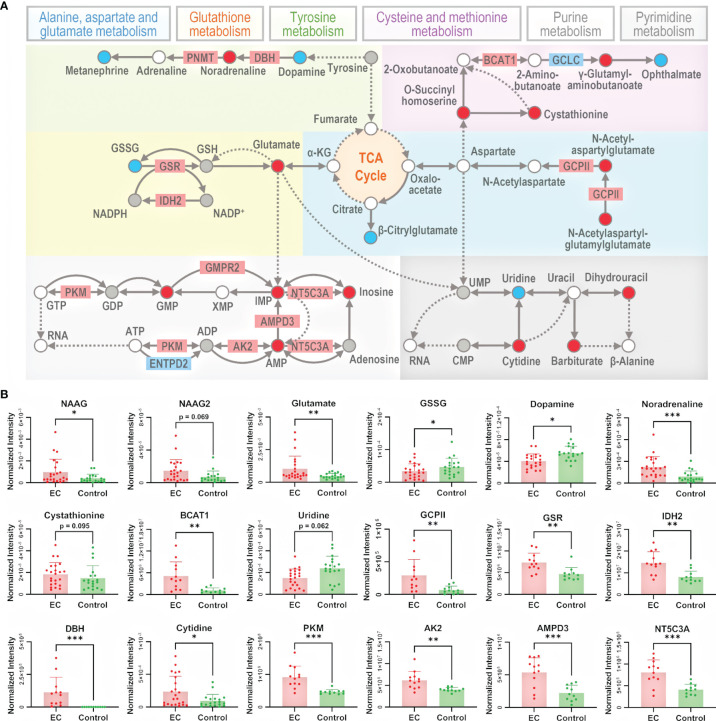
Illustration of the significant pathways and differential metabolites and proteins relating to energy metabolism connected to the TCA cycle. **(A)** Pathway overview of amino acid metabolism and nucleotide metabolism. Circles represent metabolites and rectangles represent proteins. Red-filled circles or rectangles indicate that the metabolites or proteins are upregulated in the EC group compared to the control group, and blue-filled ones indicate downregulation. Gray-filled circles represent the identified but not significantly changed metabolites, and the white-filled ones represent the unidentified metabolites to better connect and explain the pathways. Solid lines indicate direct reactions between metabolites, while dashed lines indicate multi-step reactions. **(B)** Normalized intensity of metabolites and proteins involved in the illustrated pathways. NAAG, NAAG2, GSSG, GCPII, GSR, IDH2, DBH, BCAT1, PKM, AK2, AMPD3, and NT5C3A represent N-acetylaspartylglutamate, N-acetylaspartylglutamylglutamate, glutathione disulfide, glutamate carboxypeptidase II, glutathione-disulfide reductase, isocitrate dehydrogenase 2, dopamine β-hydroxylase, branched chain amino acid transaminase 1, pyruvate kinase M1/2, adenylate kinase 2, adenosine monophosphate deaminase 3, and cytosolic 5’-nucleotidase 3A, respectively. Error bars represent the standard deviation. “*”, “**”, and “***” indicate *p*-values smaller than 0.05, 0.01, and 0.001, respectively. See also **Supplementary Figures 8**, **9** for other significant metabolites and proteins.

To better understand the metabolomic dysregulation for the pathways of amino acid metabolism and nucleotide metabolism, the interaction among differential metabolites and differential proteins was taken into consideration. Metabolic pathways are composed by reactions of metabolites catalyzed by proteins, so pathways owning direct transformation between differential metabolites or direct interaction between differential metabolites and differential proteins were chosen for further explanation. There were 13 pathways selected, i.e., alanine, aspartate, and glutamate metabolism; arginine and proline metabolism; arginine biosynthesis; tryptophan metabolism; phenylalanine, tyrosine, and tryptophan biosynthesis; phenylalanine metabolism; cysteine and methionine metabolism; beta-alanine metabolism; lysine degradation; tyrosine metabolism; glutathione metabolism; pyrimidine metabolism; and purine metabolism. To better summarize the metabolomic changes in a pathway-and-compound-based way, 6 out of the 13 selected pathways were illustrated in detail by marking up- and downregulated metabolites and proteins (**Figure 4A**). The change in specific metabolites and proteins is shown in bar plots in **Figure 4B** and **Supplementary Figures 8**, **9**. It should be noted that the six pathways are connected with the metabolites in the TCA cycle, which is an energy-relating anabolic process that can promote cancer growth (42, 44, 45). Though most of the intermediates in the TCA cycle were not detected by the untargeted metabolomic method, the linkage of the TCA cycle with many significant pathways indicated the essential role of the TCA cycle in the growth of a tumor.

In alanine, aspartate, and glutamate metabolism, N-acetylaspartylglutamate (NAAG) and N-acetylaspartylglutamylglutamate (NAAG2) were upregulated, and the enzyme glutamate carboxypeptidase II (GCPII) catalyzing the reaction from NAAG2 to NAAG as well as NAAG to N-acetylaspartate (NAA) was also upregulated. NAAG and NAAG2 are peptide-based neurotransmitters in the mammalian nervous system and are related to neuro functions (46). Besides the studies of NAAG in brain cancers like glioma (47), it has been reported that NAAG can serve as a reservoir to provide glutamate to tumor cells in cancers expressing GCPII, such as ovarian cancer, where NAAG is more abundant in more malignant tumors and its concentration in plasma is correlated with tumor size (48). Indeed, we have observed the upregulation of glutamate, which further supported that the NAAG here acted as a source of glutamate to promote cancer cell growth (49).

In glutathione metabolism, glutathione disulfide (GSSG) was downregulated, and the enzymes glutathione-disulfide reductase (GSR) and isocitrate dehydrogenase 2 (IDH2), catalyzing, respectively, the process of GSSG to glutathione (GSH) and NADP^+^ to NADPH, were upregulated. GSH is the most abundant antioxidant in living organisms, and researchers have found that an excess concentration of GSH can promote tumor progression and is correlated with increased metastasis (50). Considering the complex role of GSH in cancer metabolism, its insignificance of change in metabolome was not surprising. The dysregulation of the abovementioned metabolites and proteins indicates an endeavor of maintaining GSH in the reduced state, with the trend of converting NADP^+^ back to NADPH at the same time.

In tyrosine metabolism, dopamine was downregulated while noradrenaline was upregulated, and the enzyme dopamine β-hydroxylase (DBH) catalyzing the conversion of dopamine to noradrenaline was upregulated. Dopamine is a catecholamine associated with tumorigenesis regulation by affecting angiogenesis and cell proliferation (51), and it can lower the chance of cancer stem cell-induced apoptosis (52). On the other hand, studies have also found that noradrenaline can promote an angiometabolic switch in endothelial cells to activate tumor angiogenesis, resulting in cancer progression (53). Thus, the insufficiency of dopamine and redundancy of noradrenaline emboldened by the activated DBH can both accelerate the growth of a tumor.

In cysteine and methionine metabolism, cystathionine and branched-chain amino acid transaminase 1 (BCAT1) were both upregulated. Cystathionine is a dipeptide generated from serine and homocysteine. A study in breast cancer found that cystathionine accumulates in tissue for cancer cells to gain additional homeostatic stability to their endoplasmic reticulum and mitochondria, elevating the apoptotic threshold (54). BCAT1 catalyzes the catabolism of branched-chain amino acids (BCAAs), and the association of BCAAs with different cancer phenotypes has been demonstrated in a series of studies (55, 56). The overexpression of BCAT1 promotes tumor growth in gynecological cancers, as in ovarian cancer (57) and breast cancer (58). Therefore, the upregulation of both cystathionine and BCAT1 promotes the growth of a tumor.

Nucleotide metabolism includes the generation of purine and pyrimidine molecules for critical procedures like DNA replication, RNA synthesis, and cellular bioenergetics (59). The activation of nucleotide metabolism can promote the uncontrolled growth of a tumor. Genes and proteins relating to the process have already been considered targets of therapy (59). The dysregulation of nucleotide metabolism has been extensively studied for cancers like glioma (60) and breast cancer (61). There are reviews discussing the role of nucleotide metabolism in cancers from both the proliferative (62) and the non-proliferative (59) aspects. In this study, many metabolites and proteins involved in nucleotide metabolism were found to be significantly dysregulated.

In purine metabolism, pyruvate kinase (PK), adenylate kinase 2 (AK2), adenosine monophosphate deaminase 3 (AMPD3), and cytosolic 5’-nucleotidase 3A (NT5C3A) catalyze the 4 reactions from adenosine 5’-triphosphate (ATP) to inosine. They were all upregulated, and AMP, inosine 5’-monophosphate (IMP), and inosine on this reaction chain were upregulated as well, indicating the activation of the whole pathway of purine metabolism. The sequence of conversion further relates to the synthesis of RNA and DNA. It has been reported that purine metabolism could be involved in tumor myometrial invasion of EC (63). PK has two isoforms, PKM1 and PKM2. PKM1 expression following PKM2 loss can cause the proliferation arrest of primary cells and alter nucleotide synthesis, which can influence cell growth (64). AK2 catalyzes the reaction of nucleotide phosphorylation (65). Its localization in mitochondrial intermembrane suggests a unique role of the enzyme in energy metabolism (66). Recent studies provided evidence that AK2 is overexpressed in lung adenocarcinoma, and is associated with tumor progression (67). AK2 has the potential of being a radiosensitive biomarker to predict the toxicity of radiotherapy to normal tissue (68). AMPD3 catalyzes the hydrolytic deamination of AMP to form IMP (69), whose overexpression is associated with the malignant characteristics of gastrointestinal stromal tumors (70). AMPD3 also showed a significantly enhanced level in prostate tumor tissue, indicating high oxidative stress and frequent transformation of nucleotides to nucleosides (71).

In pyrimidine metabolism, despite an absence of significantly differential proteins, the change of metabolism could also be seen from the dysregulation of metabolites. Cytidine deaminase (CDA) catalyzes the hydrolytic deamination of cytidine to uridine (72), and it has been proven that CDA deficiency leads to DNA damage (73), associating with cancer development (74). The upregulation of cytidine and downregulation of uridine in this study suggests a lack of CDA.

### Verification of the Significant Pathways Using Urine and Intrauterine Brushing Samples

Since the collection of tissue samples cannot avoid invasive procedures like biopsy, hysteroscopy, or surgery, there is a high demand for diagnosis methods with non-invasive or minimally invasive sampling. Urine and intrauterine brushing samples can be collected in a non-invasive or minimally invasive way. The significant pathways identified using tissue samples were then verified by the metabolomic analysis of urine and intrauterine brushing samples, focusing on the metabolites relating to the pathways of amino acid metabolism and nucleotide metabolism. Urine samples were obtained from another 10 patients with EC and another 12 controls. Intrauterine brushing samples were obtained from 10 patients with EC and 11 controls, different from the donors of tissue and urine samples. Untargeted metabolomic analysis, data processing, and structure identification were done in the same way as for the tissue samples. TICs of QC samples (**Supplementary Figures 10A**, **11A**) showed a good overlap, demonstrating the stability and repeatability of the measuring system. A total of 8,066 features were obtained for the urine samples with 349 differential metabolites (*p*-values < 0.05 or VIP values > 1) being structurally identified. A total of 4,296 features were obtained for the intrauterine brushing samples with 93 differential metabolites (*p*-values < 0.05 or VIP values > 1) being structurally identified. Statistical analysis based on features, including PCA score plots, PLS-DA score plots, and volcano plots, is shown in **Supplementary Figures 10**, **11**. From the volcano plots, there is a trend of general downregulation of urine metabolites in EC patients.

**Figure 5:**
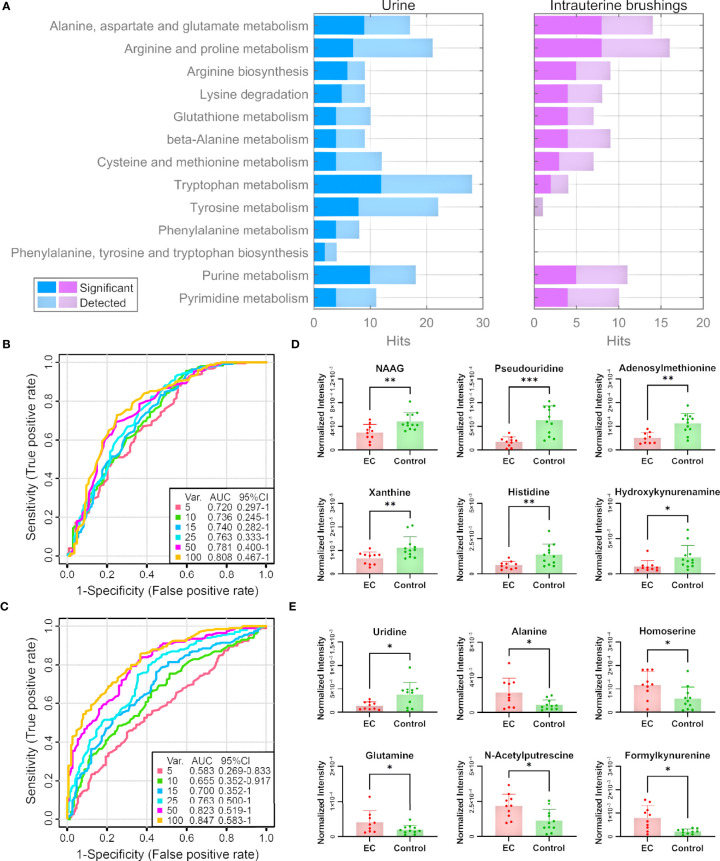
Pathway and biomarker analyses with EC urine and intrauterine brushing samples based on the 13 selected pathways by the analysis of EC tissue samples. **(A)** Numbers of detected and significant metabolites (*p*-values < 0.05 or VIP values > 1) from the urine and intrauterine brushing samples relating to the 13 selected pathways. **(B, C)** ROC curves for the classification of EC patients and controls using the metabolites detected in **(B)** urine and **(C)** intrauterine brushing samples relating to the 13 selected pathways. PLS-DA was used as the classification method, and 100 rounds of Monte Carlo cross-validation were performed to generate the ROC curve. Details are described in the “Materials and Methods” section. **(D, E)** The normalized intensity of representative metabolites detected in **(D)** urine and **(E)** intrauterine brushing samples relating to the 13 selected pathways. NAAG represents N-acetylaspartylglutamate. “*”, “**”, and “***” indicate *p*-values smaller than 0.05, 0.01, and 0.001, respectively. See also **Supplementary Figures 12**, **13** for other significant metabolites.

To verify the 13 selected pathways by the analysis of tissue samples, we focus on the metabolites identified from the urine and intrauterine brushing samples related to the 13 pathways. The lists of 285 urine metabolites and 122 intrauterine brushing metabolites are shown in **Supplementary Dataset 3**. For each pathway of both biosamples, the numbers of metabolites that were detected and significantly regulated between the EC and control groups (*p*-values < 0.05 or VIP values > 1) are shown in **Figure 5A** and **Supplementary Table 2**. Metabolites of all the 13 pathways were also highly identified and altered for the urine EC samples, and 10 out of 13 for the intrauterine brushing EC samples.

We then assessed whether the 285 urine metabolites and the 122 intrauterine brushing metabolites relating to the 13 pathways could include potential biomarkers for the classification of EC patients and controls. PLS-DA was used as the classification method. Cross-validations (described in the “Materials and Methods” section) were performed to generate the receiver operating characteristic (ROC) curves (**Figures 5B, C**). For the urine samples, the highest area under the curve (AUC) was 0.808 with the top 100 selected metabolites, but the AUC values did not change much when changing the number of selected top metabolites from 5 to 100 (**Figure 5B**). The results indicated that urine metabolites relating to the 13 pathways selected by the analysis of tissue samples can serve as potential biomarkers for the identification of EC, and the models based on top several significant metabolites showed good classification performance. Some of the significant urine metabolites, which were selected most frequently (among the top 15, frequency ≥ 0.94) during the 100-feature-model based cross-validation, are shown in the bar charts (**Figure 5D**). Most of the significant metabolites were downregulated in the urine samples of EC patients compared to the controls.

For the intrauterine brushing samples, the AUC values increased with the number of selected metabolites and reached the highest AUC value of 0.847 with 100 selected metabolites (**Figure 5C**). The result indicated that for intrauterine brushing samples, limited metabolites (5–25) were not sufficient for building classification models because of the fluctuation of different metabolites. Some of the significant intrauterine brushing metabolites, which were selected most frequently (among top 25, frequency = 1.0) during the 100-feature-model-based cross-validation, are shown in the bar charts (**Figure 5E**).

**Figure 6:**
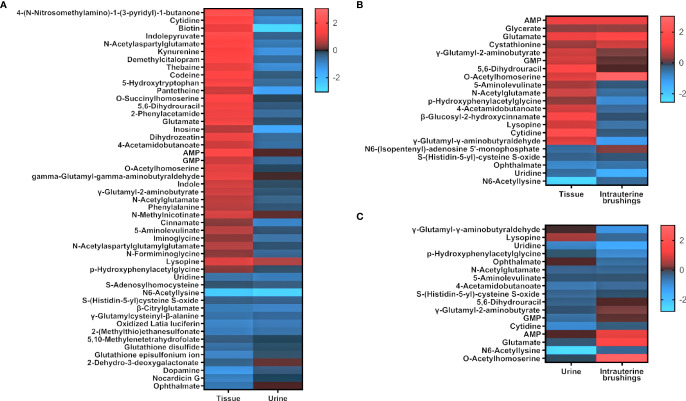
The regulation of significant metabolites identified from tissue samples compared to those in urine and intrauterine brushing samples. Heatmaps show the comparison of log_2_(FC) values (EC patients to controls) between **(A)** tissue and urine, **(B)** tissue and intrauterine brushings, and **(C)** urine and intrauterine brushings. Red indicates upregulation; blue indicates downregulation; dark indicates no change.

To compare the metabolomic changes in tissue, urine, and intrauterine brushings, the 74 significant metabolites identified from tissue samples were chosen, and the regulations of the metabolites were compared among the three types of samples. The log_2_(FC) values between the EC patients and controls are shown as a heatmap in **Figure 6**. Among the 74 metabolites, 47 were upregulated and 27 were downregulated in the tissue samples. Forty-nine of them were also detected and identified in urine samples, with 6 upregulated and 43 downregulated, showing, in general, an opposite trend compared to the tissue metabolites. Twenty-one of the 74 metabolites were detected in the intrauterine brushing samples, with 9 upregulated and 12 downregulated, which showed a generally consistent trend compared to the tissue metabolites.

## Discussion

Metabolomic analysis is an increasingly attractive approach to researching EC (27–29). Researchers have focussed on the diagnosis of EC (9–14, 16, 18–26), the differentiation of EC stages (10, 18, 20, 22), the influence of risk factors (13, 15–17), and the possible pathogenesis of tumor development (9–14, 17–22, 24–26) using metabolomic methods. These studies have provided massive information for EC, like the alteration of metabolomics, the establishment of possible diagnosis models, and the enrichment of critical metabolism pathways. Every single study can provide pieces of enlightening results. However, limited correlation among the different studies can be identified, and sometimes contradiction may even be found. This is mainly due to the diversified sample collection and measurement strategies used in different studies. Thus, there are results like alanine, leucine, tyrosine, and valine upregulated in tissue (9) but downregulated in serum (22), and serine upregulated according to GC-MS-based analysis (22) but downregulated according to NMR-based analysis (18) in the metabolomics study of EC. In addition, as one metabolite can be involved in multiple reactions, the dysregulation of a specific metabolite can be a result of several different processes. Therefore, it is difficult to identify the real altered metabolic pathways in EC solely by metabolomic analysis.

In this work, we applied multi-omics analyses to multiple types of biosamples, aiming at exploring the metabolomic change of EC in a pathway-based instead of metabolite-based way. To gain a more reasonable illustration of this view, the patients selected for the EC group were all women suffering from type I endometrial carcinoma, including endometrial carcinoma G1 and G2. Since type I EC is correlated with prolonged estrogen exposure and does not have progesterone protection, it has been proved to be sensitive to the change in metabolism (75).

Starting with the integration of metabolomic and proteomic analysis of tissue samples, we take advantage of the fact that proteins catalyze chemical reactions among metabolites and embody information of genes. While metabolites function as the substrates or products of metabolomic reactions, the regulation of proteins can provide clear information on the activation or inactivation of metabolomic reactions. Blending the alteration of proteins into the network of metabolites, a more evident map of changes in metabolomic pathways can be obtained. Herein, by integrating the proteomic and metabolomic analysis, significant pathways of amino acid metabolism and nucleotide metabolism were revealed. Meanwhile, the network illustration connecting pathways of amino acid metabolism and nucleotide metabolism not only provides a possible explanation for energy metabolism in EC but also offsets some shortcomings of metabolomic measurement. A previous review has pointed out that the downregulation of amino acids can be a signal of EC, but the changes in amino acids are not significant (28). Although no significantly changed amino acids were identified in the tissue samples in this work, the network illustration shows that the amino acid metabolism pathways are significantly changed and can be alternatives to the amino acid themselves as biomarkers of EC.

Based on the proteomic and metabolomic analysis results of tissue samples, we moved forward to the metabolomics of urine and intrauterine brushings. Non-invasive diagnosis by urine and vaginal samples has been reported (76). Urine collection is non-invasive but research on EC urine metabolomics is still limited (25). Using special brushes to collect intrauterine fluids is a minimally invasive method now widely used in clinical diagnosis (77, 78), but to date, there is no metabolomics study on intrauterine brushings for EC. Non-invasive or minimally invasive sample collection strategies for disease diagnosis are an undeniable future trend, but the theoretical and experimental foundation is indispensable. In contrast to diagnosis by videography or pathology, the “invisibility” of metabolomics prompts the method to require more evidence and verification before clinical usage. Compared with tissues and intrauterine brushings, which can be regarded as “*in situ*” collected tumor-related samples, urine contains additional metabolomic information of other organs, such as the metabolic process in the kidney and bladder, wherein the possibility of EC affecting the functions of the organs cannot be excluded in the urine-based metabolomics study of EC (79).

The metabolomic analysis of urine and intrauterine brushings are not only a verification of the results obtained with the tissue samples, they also preliminarily demonstrate the feasibility of EC diagnosis using urine or intrauterine brushing metabolites. Monte Carlo cross-validation (MCCV) was performed for the identification of EC using the models built with urine or intrauterine brushing metabolites, while the metabolites were selected based on the 13 significant pathways, relating to the amino acid metabolism and nucleotide metabolism, suggested by the analysis of tissue samples. The results again proved the significance of the 13 pathways in urine and intrauterine brushing samples. It should be noted that since the metabolites for building the classification models were not directly chosen by machine learning from the metabolomic data of urine and intrauterine brushings, the models were not optimized in distinguishing EC patients and controls. For minimal and non-invasive diagnosis of EC, future work is needed for the metabolomic study of large cohorts of intrauterine brushings and urine samples to find biomarkers and build reliable classification models.

The comparison of the FC values (EC/control) of significant metabolites among the samples of tissue, urine, and intrauterine brushings showed that many metabolites were regulated in opposite ways in tissue and urine, while most of the metabolites kept a consistent regulation trend in tissue and intrauterine brushings. Intrauterine brushings are more closely related to tissue, while urine is a biofluid reflecting very downstream metabolism after the treatment of several organs (80, 81). The opposite trend could also be related to a hypothesis that the abnormal metabolism in a tumor may result in the accumulation of metabolites in lesions, thus decreasing their concentrations in urine. In this study, results were limited because different cohorts were involved in the metabolomic analysis of tissue, urine, and intrauterine brushing samples, making it difficult to compare in a paired way. Nevertheless, all the analyses demonstrated the significance of amino acid metabolism and nucleotide metabolism in EC, which again strengthen the conclusion.

In summary, this study demonstrated the important roles of amino acid metabolism and nucleotide metabolism in EC using multi-biosamples, illustrating the network interaction between metabolites and proteins, as well as among pathways. We also provided supporting evidence for the non-invasive or minimally invasive diagnosis of EC using urine and intrauterine brushing samples with metabolomic analysis. We expect that more comprehensive multi-omics analyses will be applied to the study of EC to further explore the mechanism and that simpler but effective diagnostic methods can be developed based on further research on multi-biosamples.

## Data Availability Statement

Proteomics data have been deposited to ProteomeXchange *via* the iProX (82) partner repository with the dataset identifiers PXD030222 and IPX0003827000. Metabolomics data have been deposited to the EMBL-EBI MetaboLights database (83) with the identifier MTBLS3935. The complete dataset can be accessed here: https://www.ebi.ac.uk/metabolights/MTBLS3935.

## Ethics Statement

The studies involving human participants were reviewed and approved by The Ethics Committee of Obstetrics and Gynecology Hospital of Fudan University. The patients/participants provided their written informed consent to participate in this study.

## Author Contributions

RY did the majority of the experiment and wrote the first draft of the manuscript. LX collected all the clinical samples. XW and CS performed the proteomic analysis and the proteome data analysis. XC and LQ designed the work and acquired the funding for the project. LQ supervised all aspects of the work and prepared the final manuscript. All authors contributed to the article and approved the submitted version.

## Funding

This research was funded by the National Natural Science Foundation of China (NSFC, 22022401, 22074022, and 21934001), the Ministry of Science and Technology of China (2020YFF0304502 and 2020YFF0426500), and the National Key Research and Development Program of China (2019YFC1005200 and 2019YFC1005201).

## Conflict of Interest

Authors XW and CS were employed by Shanghai Omicsolution Co., Ltd.

The remaining authors declare that the research was conducted in the absence of any commercial or financial relationships that could be construed as a potential conflict of interest.

## Publisher’s Note

All claims expressed in this article are solely those of the authors and do not necessarily represent those of their affiliated organizations, or those of the publisher, the editors and the reviewers. Any product that may be evaluated in this article, or claim that may be made by its manufacturer, is not guaranteed or endorsed by the publisher.
