# Supplementary material for: Multi-Omic Profiling of Multi-Biosamples Reveals the Role of Amino Acid and Nucleotide Metabolism in Endometrial Cancer
Source: Front Oncol. 2022 Apr 29;12:861142. doi: 10.3389/fonc.2022.861142 (PMC9099206; doi:10.3389/fonc.2022.861142)
Supplement: Supplementary file 1 [file DataSheet_1.zip › Supplementary_Material.pdf]

Supplementary Material

1 Supplementary Data

Supplementary Dataset 1: Differential metabolites between EC patients and controls identified from tissue samples.

Supplementary Dataset 2: Differential proteins between EC patients and controls identified from tissue samples.

Supplementary Dataset 3: Metabolites detected in urine and intrauterine brushings samples relating to the 13 pathways selected by the analysis of tissue samples.

2 Supplementary Figures and Tables

2.1 Supplementary Figures

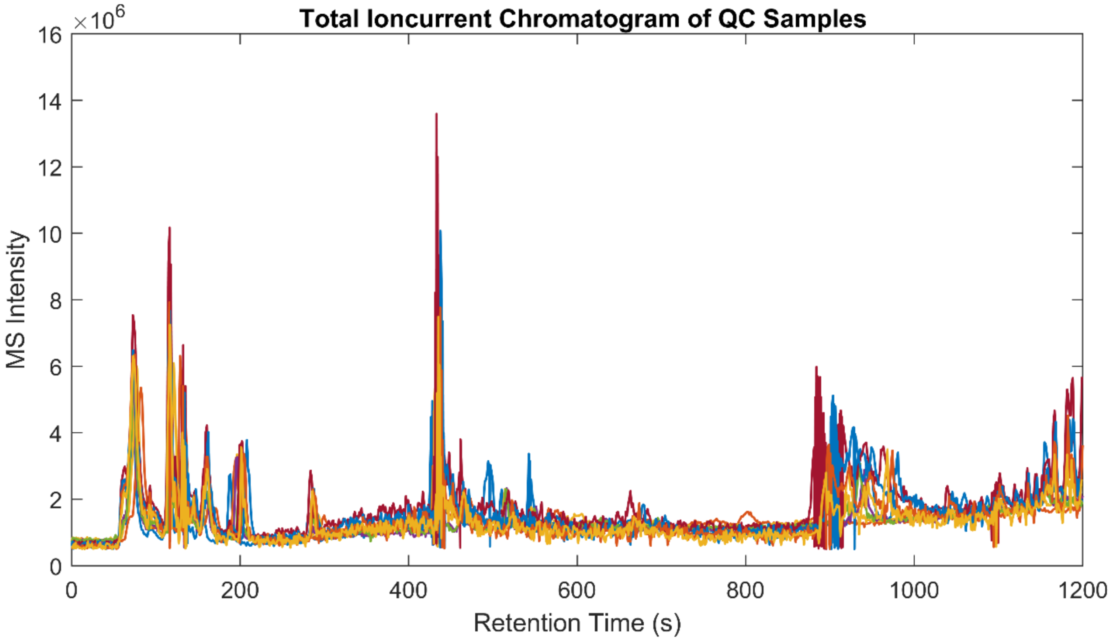

Supplementary Figure 1. TICs of QC samples for the metabolomic analysis of tissue samples.

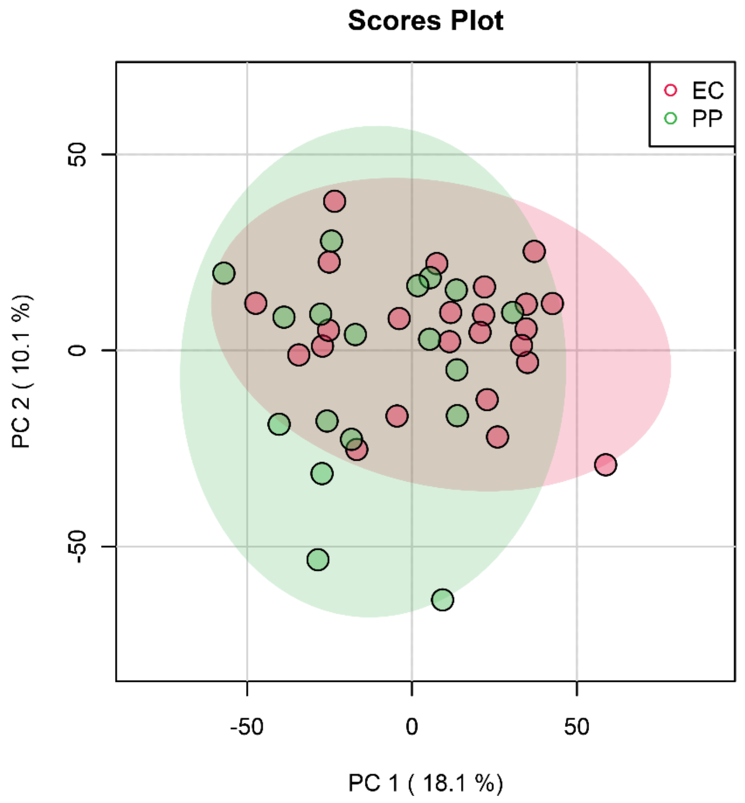

Supplementary Figure 2. PCA scores plot for the metabolomic data of tissue samples collected for EC patients and controls by LC-MS/MS in the positive ion mode.

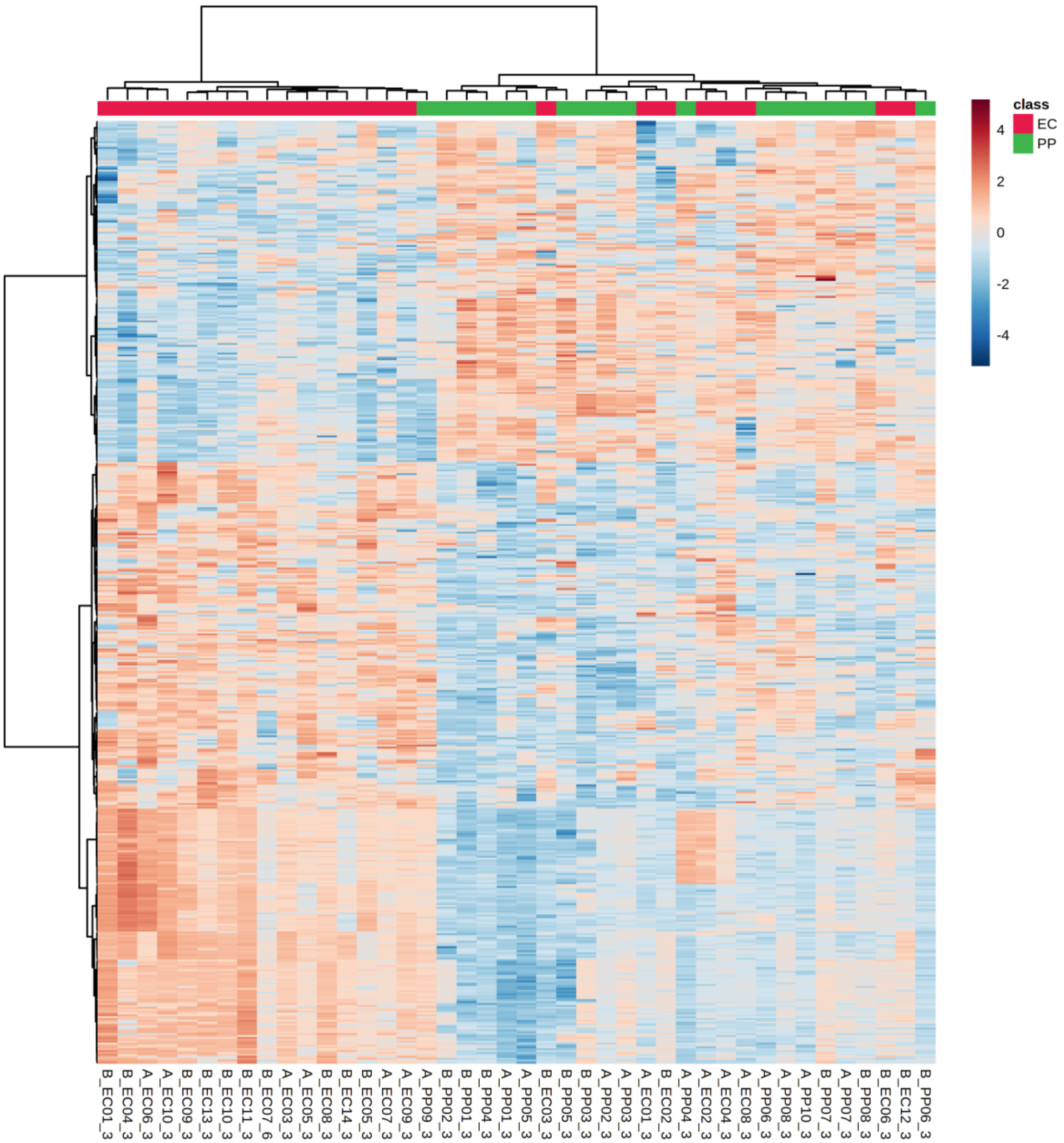

Supplementary Figure 3. Hierarchical clustering heatmap for the metabolomic data of tissue samples collected from EC patients and controls using the top 500 features with the smallest p values. Color of cells shows the transformed intensity of features according to the color scale bar.

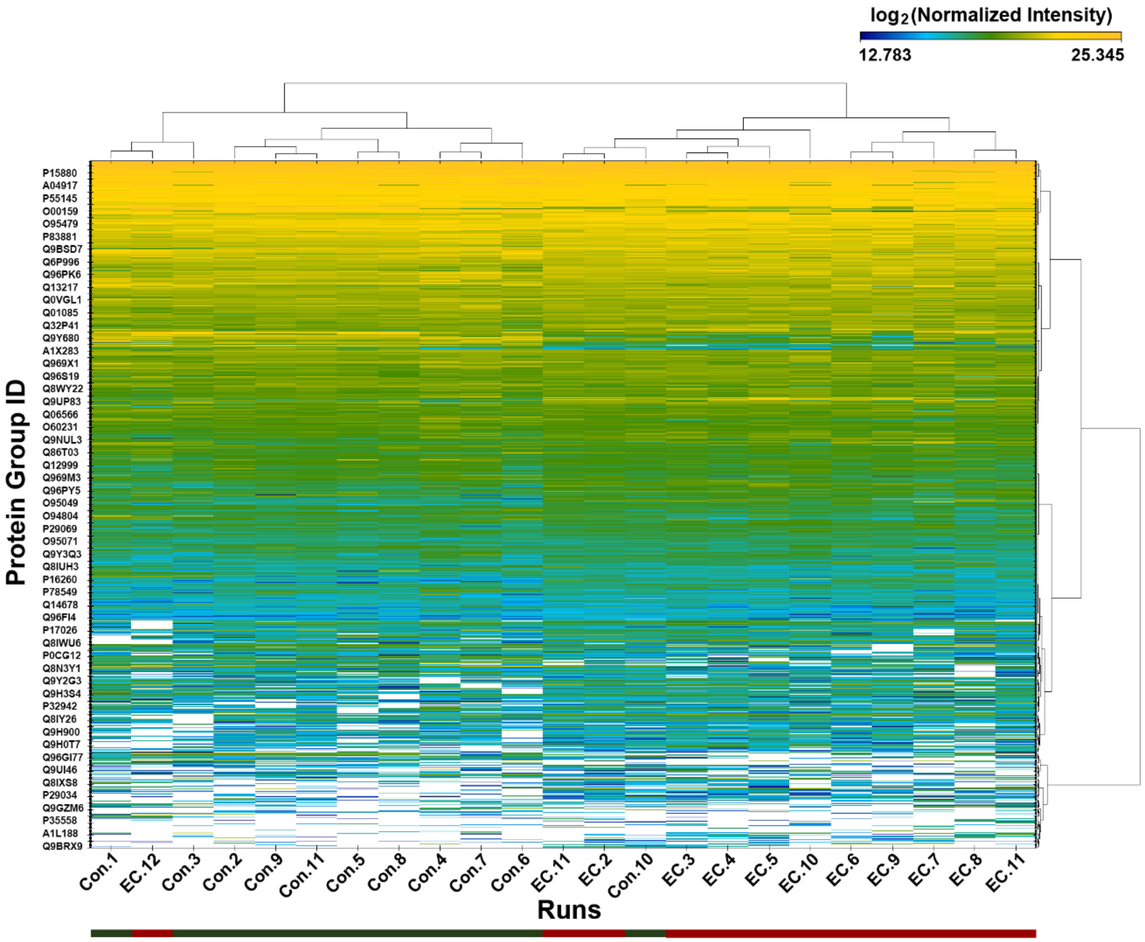

Supplementary Figure 4. Heatmap of proteomic data of tissue samples collected for EC patients and controls. Proteins were ranked from high quantity (yellow) to low quantity (blue), while white stands for missing values. In the dimension of samples, hierarchical clustering analysis (HCA) was performed to group the samples according to the similarity of protein expression.

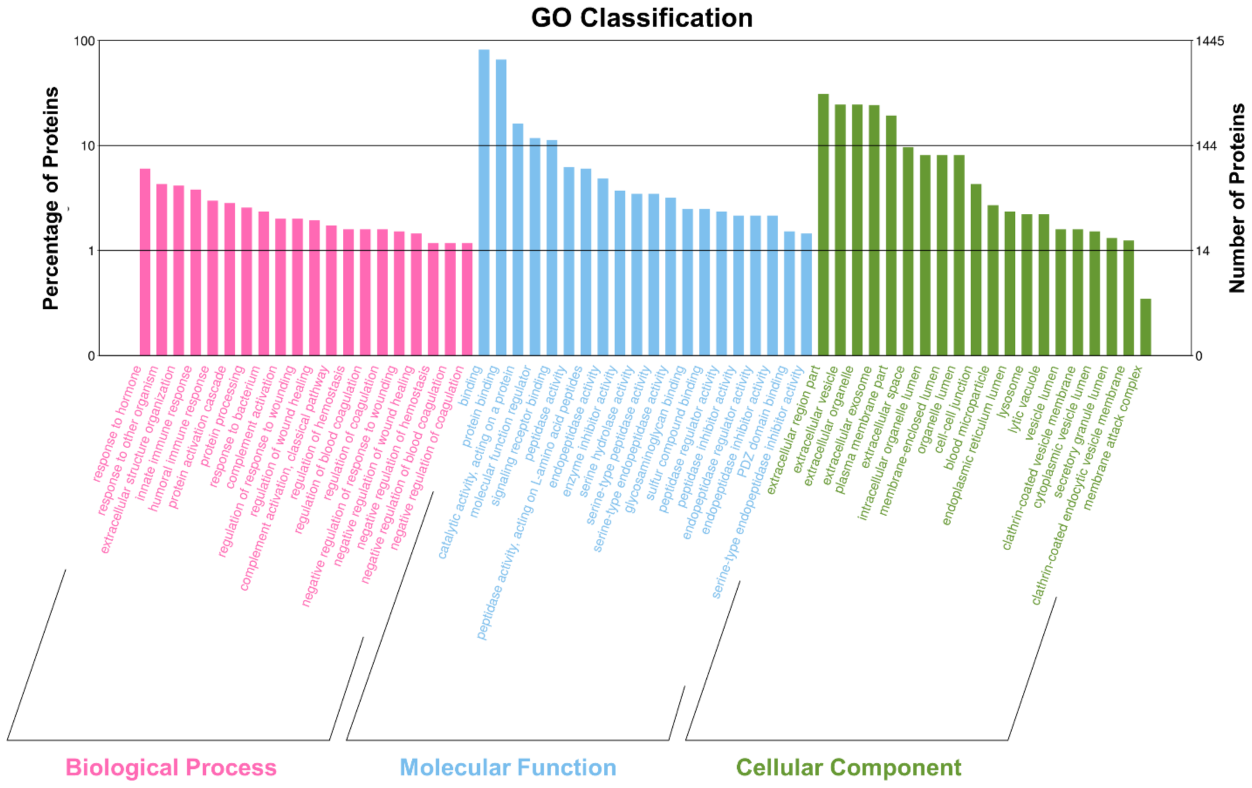

Supplementary Figure 5. GO classification plot of the differential proteins in EC tissue samples compared to controls. The 1445 differential proteins were annotated and classified by the gene names into three domains called biological process (pink), molecular function (blue) and cellular component (green). The 20 most significant terms with smallest p values of each domain were plotted. The horizontal axis shows the GO terms with their domains. The vertical axes show the number of proteins annotated in each term on the right and its percentage on the left.

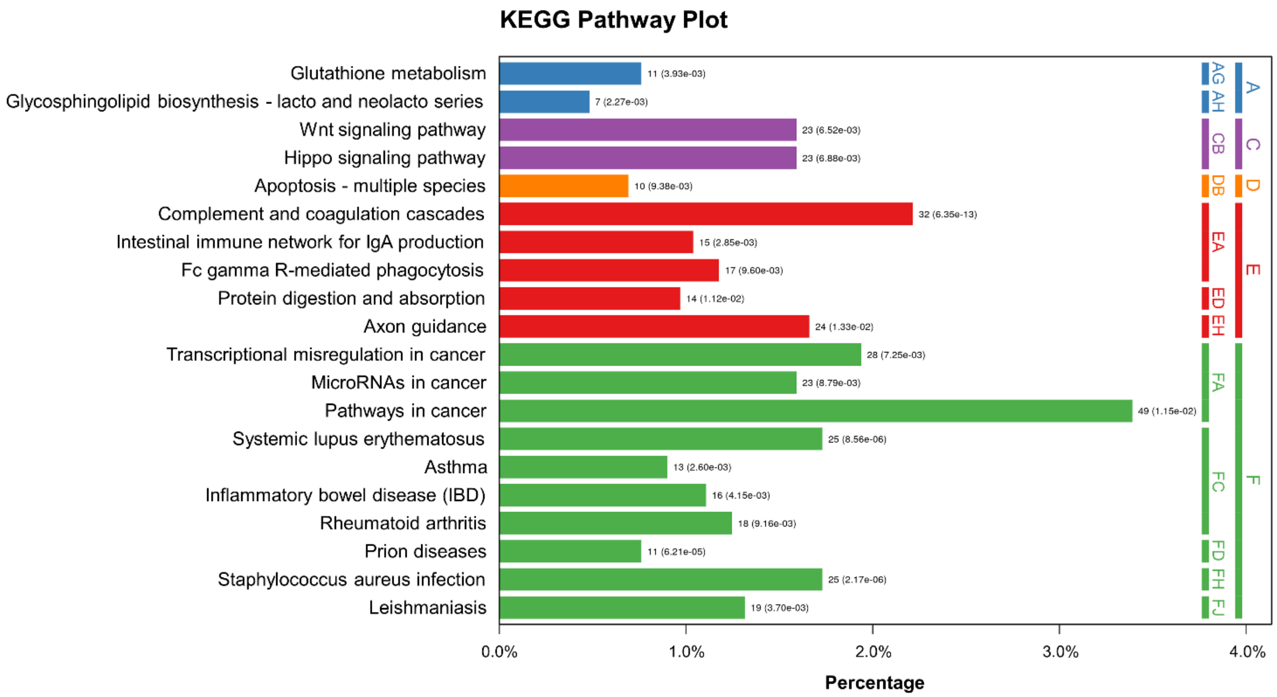

Supplementary Figure 6. KEGG pathway enrichment analysis based on the differential proteins in EC tissue samples compared to controls. The 1445 differential proteins were enriched in some of the KEGG pathways, and the 20 most significant pathways with smallest p values were plotted. The horizontal axis shows percentage of proteins enriched in each pathway. The vertical axes show the pathway names on the left and their categories on the right. The category represents by each alphabet abbreviation is as follows: A, Metabolism; C, Environmental Information Processing; D, Cellular Processes; E, Organismal Systems; F, Human Diseases; AG, Metabolism of other amino acids; AH, Glycan biosynthesis and metabolism; CB, Signal transduction; DB, Cell growth and death; EA, Immune system; ED, Digestive system; EH, Development; FA, Cancers, Overview; FC, Immune diseases; FD, Neurodegenerative diseases; FH, Infectious diseases, Bacterial; and FJ, Infectious diseases, Parasitic.

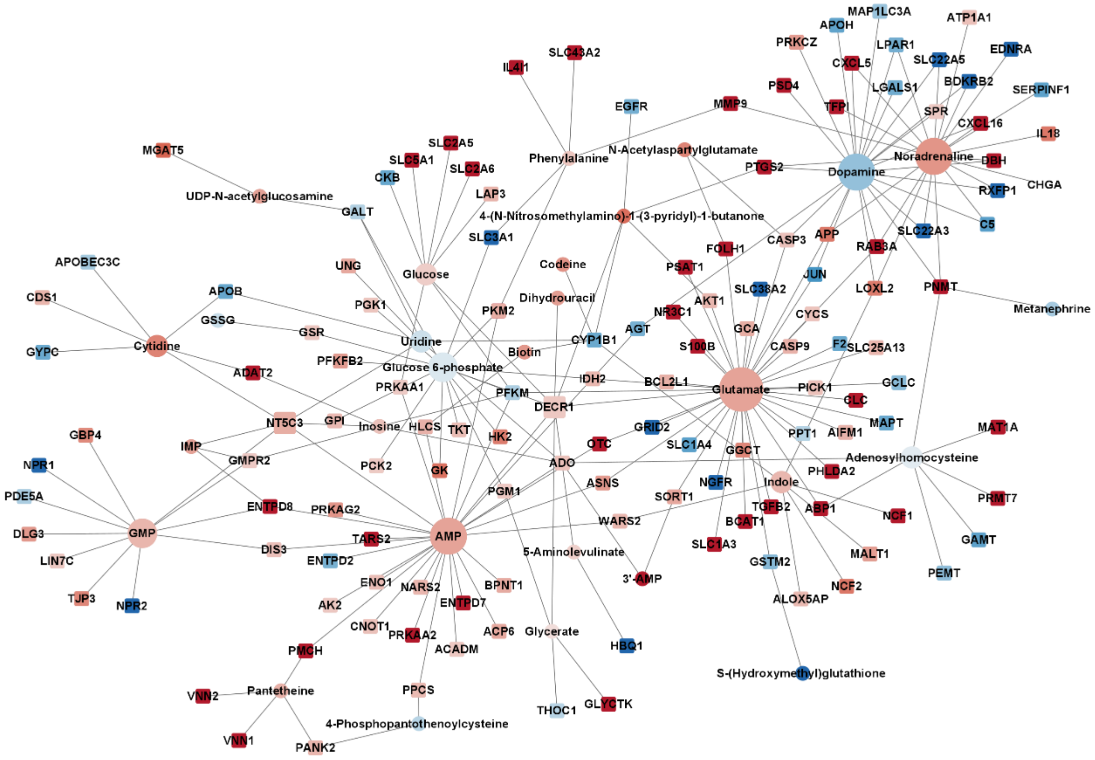

Supplementary Figure 7. Network view for the interaction between differential metabolites and differential proteins of EC tissue samples generated based on the associations in STITCH. Circles represent for metabolites and squares for proteins. Size of circles and squares indicate the number of edges connected to each node. Red represents for up-regulation and blue for down-regulation in EC patients compared to controls.

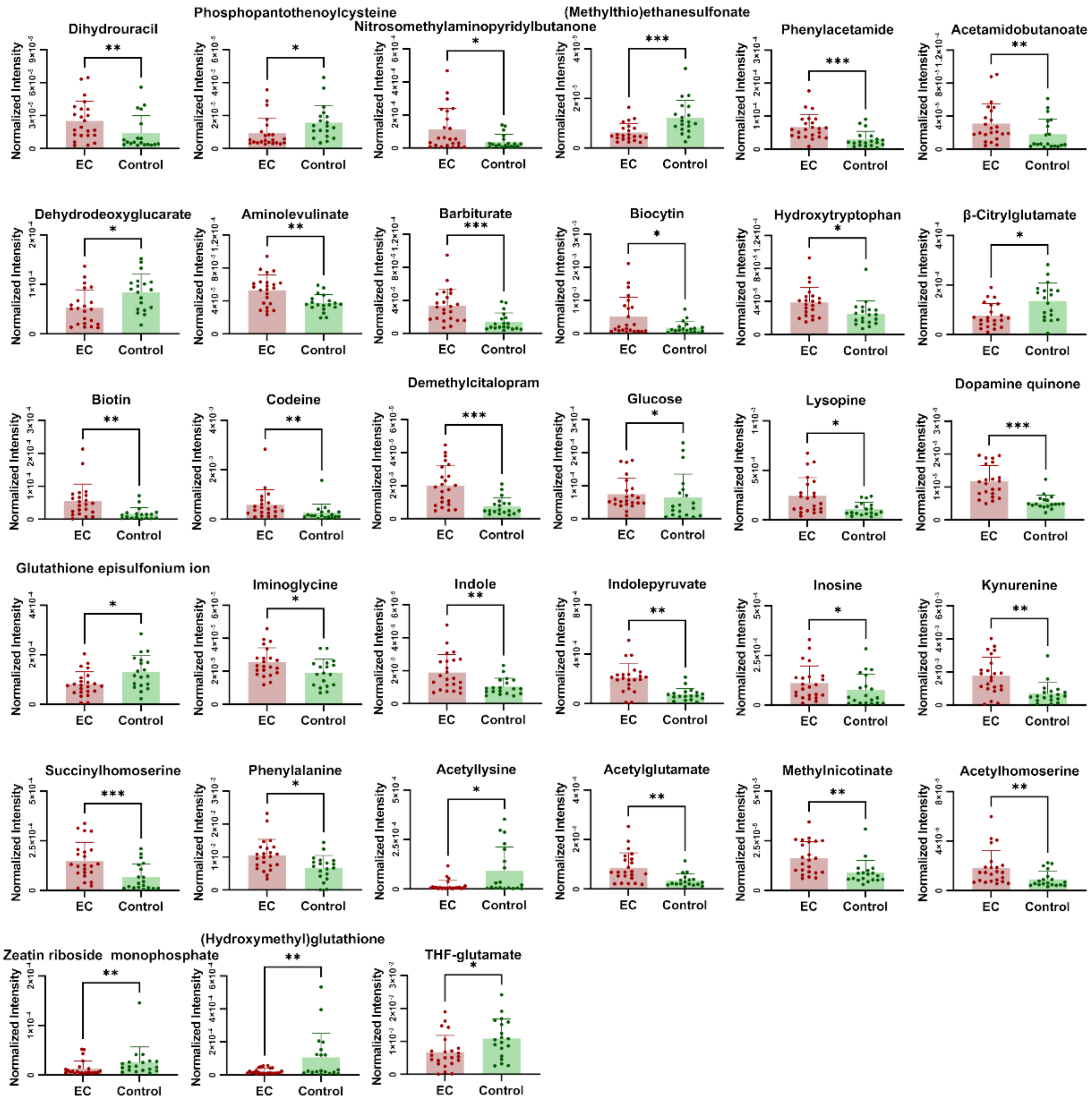

Supplementary Figure 8. Some differential metabolites identified from tissue samples. Error bars represent the standard deviation. “*”, “**” and “***” indicate p values smaller than 0.05, 0.01 and 0.001, respectively.

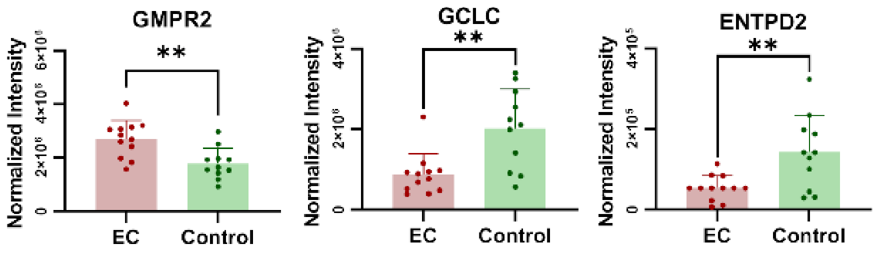

Supplementary Figure 9. Some differential proteins identified from tissue samples. GMPR2, GCLC and ENTPD2 represent guanosine monophosphate reductase 2, glutamate-cysteine ligase catalytic subunit and ectonucleoside triphosphate diphosphohydrolase 2, respectively. Error bars represent the standard deviation. “*”, “**” and “***” indicate p values smaller than 0.05, 0.01 and 0.001, respectively.

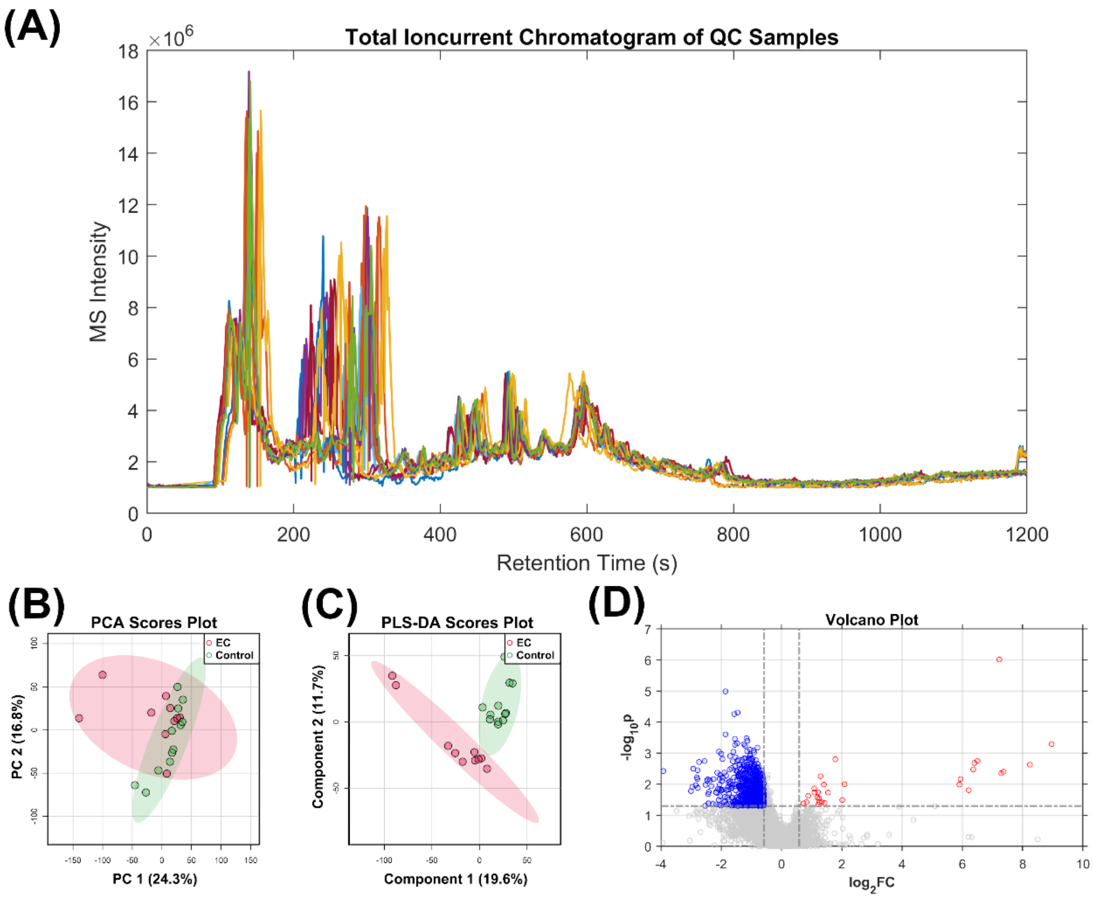

Supplementary Figure 10. TICs of QC samples and statistical analysis results of metabolomic data of urine samples. **(A)** TICs of QC samples for the metabolomic measurement of urine samples. **(B)** PCA scores plot and **(C)** PLS-DA scores plot for the metabolomic data of urine samples collected for EC patients and controls by LC-MS/MS in the positive ion mode. **(D)** Volcano plots of urine metabolites features with $\log_2$(FC) as the horizontal axis and $-\log_{10}$(p value) as the vertical axis. FC, fold change of EC to control.

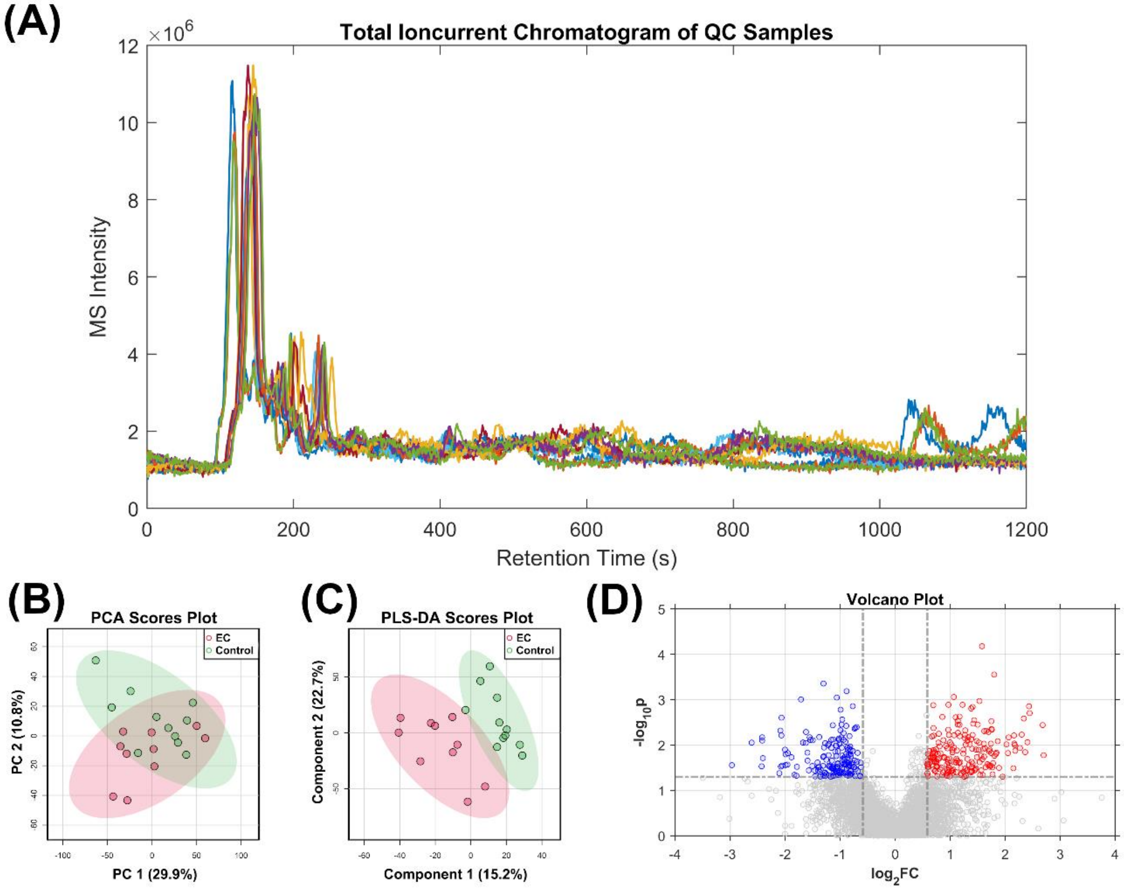

Supplementary Figure 11. TICs of QC samples and statistical analysis results of metabolomic data of intrauterine brushings samples. **(A)** TICs of QC samples for the metabolomic measurement of intrauterine brushings samples. **(B)** PCA scores plot and **(C)** PLS-DA scores plot for metabolomic data of intrauterine brushing samples collected for EC patients and controls by LC-MS/MS in the positive ion mode. **(D)** Volcano plots of intrauterine brushings metabolites features with $\log_2$(FC) as the horizontal axis and $-\log_{10}$(p value) as the vertical axis. FC, fold change of EC to control.

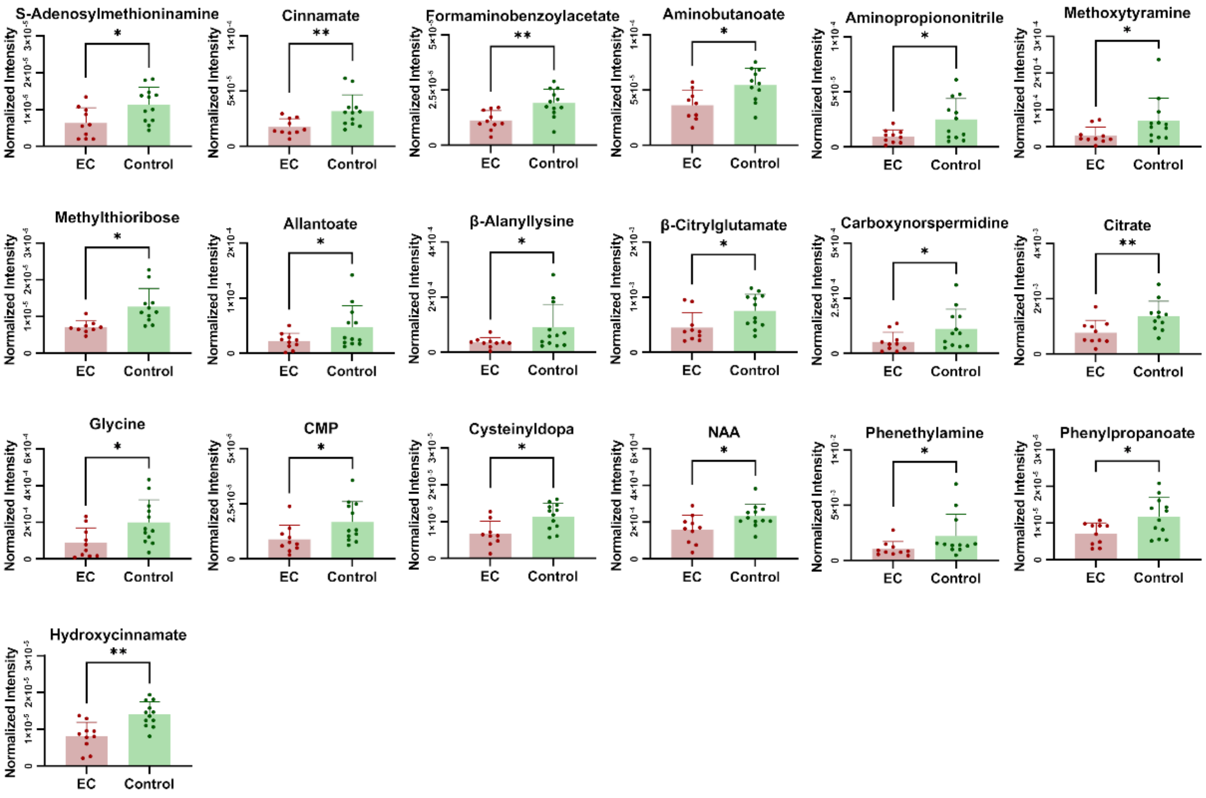

Supplementary Figure 12. Some differential metabolites identified from urine samples. CMP and NAA represent cytidine-5'-monophosphate and N-Acetylaspartate, respectively. Error bars represent the standard deviation. “*”, “**” and “***” indicate p values smaller than 0.05, 0.01 and 0.001, respectively.

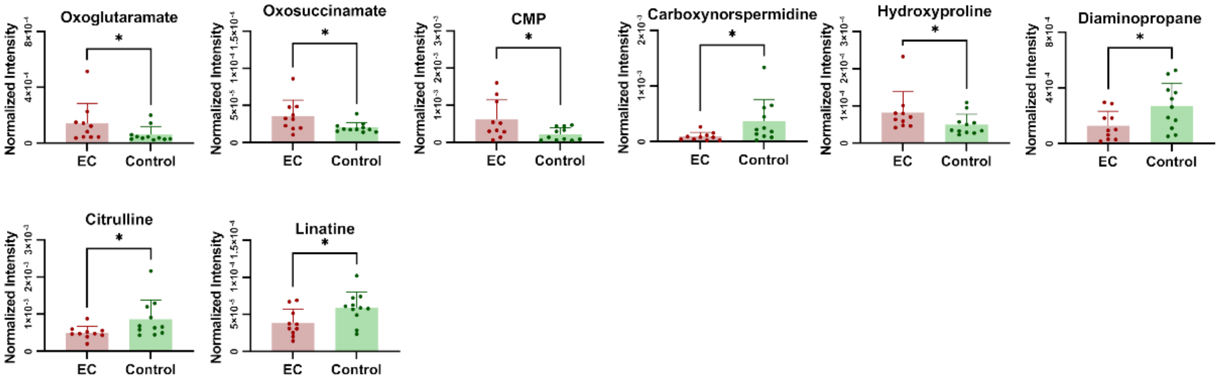

Supplementary Figure 13. Some differential metabolites identified from intrauterine brushings samples. CMP represents cytidine-5'-monophosphate. Error bars represent the standard deviation. “*”, “**” and “***” indicate p values smaller than 0.05, 0.01 and 0.001, respectively.

2.2 Supplementary Tables

Supplementary Table 1. Patients information

Parameter \ Recruitment		Tissue metabolomics		Tissue proteomics		Urine metabolomics		Intrauterine brushings metabolomics	
		**EC**	**Control**	**EC**	**Control**	**EC**	**Control**	**EC**	**Control**
Number of cases		24	18	12	11	10	12	10	11
Age (Mean ± SD) [years]		48.2±13.0	39.8±5.3	47.2±13.8	43.0±5.0	44.0±11.6	44.3±9.1	45.8±9.9	48.2±5.7
BMI (Mean ± SD) [kg/m^2]		24.8±3.9	23.0±2.5	25.7±5.1	22.9±2.2	27.1±5.9	22.4±2.9	24.8±3.6	22.6±3.0
Postmenopausal women [n(%)]		8 (33.3)	0 (0)	5 (41.7)	1 (9.1)	3 (30.0)	3 (25.0)	2 (20.0)	2 (18.0)
Previous full-term pregnancy [n(%)]		18 (75.0)	15 (83.3)	9 (75.0)	10 (90.9)	5 (50.0)	12 (100)	8 (80.0)	10 (100)
Diabetes [n(%)]		0 (0)	0 (0)	0 (0)	0 (0)	0 (0)	0 (0)	1 (10.0)	1 (9.1)
Hypertension [n(%)]		2 (8.3)	51 (5.6)	1 (8.3)	2 (18.2)	1 (10.0)	2 (16.7)	2 (20.0)	3 (27.3)
Smoking history [n(%)]		0 (0)	0 (0)	0 (0)	0 (0)	0 (0)	0 (0)	1 (10.0)	0 (0)
HRT history [n(%)]		7 (29.2)	4 (22.2)	4 (33.3)	2 (18.2)	1 (10.0)	0 (0)	1 (10.0)	2 (18.2)
Grade	G1 [n(%)]	24 (100)	-	12 (100)	-	9 (90.0)	-	9 (90.0)	-
	G2 [n(%)]	0 (0)	-	0 (0)	-	1 (10.0)	-	1 (10.0)	-
FIGO Stage	IA [n(%)]	22 (91.7)	-	11 (91.7)	-	10 (100)	-	10 (100)	-
	II [n(%)]	1 (4.2)	-	1 (8.3)	-	0 (0)	-	0 (0)	-
	IIIA [n(%)]	1 (4.2)	-	0 (0)	-	0 (0)	-	0 (0)	-

HRT represents hormone replacement therapy

Supplementary Table 2. Numbers of detected and significant metabolites (p values < 0.05 or VIP values > 1) from the urine and intrauterine brushings samples relating to the 13 selected pathways.

NO.	KEGG ID	Pathway name	Urine metabolome data		Intrauterine brushings metabolome data	
			Detected	Significant	Detected	Significant
1	hsa00250	Alanine, aspartate and glutamate metabolism	17	9	14	8
2	hsa00330	Arginine and proline metabolism	21	7	16	8
3	hsa00220	Arginine biosynthesis	9	6	9	5
4	hsa00310	Lysine degradation	9	5	8	4
5	hsa00480	Glutathione metabolism	10	4	7	4
6	hsa00410	beta-Alanine metabolism	9	4	9	4
7	hsa00270	Cysteine and methionine metabolism	12	4	7	3
8	hsa00380	Tryptophan metabolism	28	12	4	2
9	hsa00350	Tyrosine metabolism	22	8	1	0
10	hsa00360	Phenylalanine metabolism	8	4	0	0
11	hsa00400	Phenylalanine, tyrosine and tryptophan biosynthesis	4	2	0	0
12	hsa00230	Purine metabolism	18	10	11	5
13	hsa00240	Pyrimidine metabolism	11	4	10	4
